# Cognition and education benefits of increased hemoglobin and blood oxygenation in children with sickle cell disease

**DOI:** 10.1371/journal.pone.0289642

**Published:** 2023-08-08

**Authors:** Joanna P. MacEwan, Allison A. King, Andy Nguyen, Anuj Mubayi, Irene Agodoa, Kim Smith-Whitley

**Affiliations:** 1 PRECISIONheor, Los Angeles, California, United States of America; 2 Division of Pediatric Hematology/Oncology, Washington University and St. Louis Children’s Hospital, St. Louis, Missouri, United States of America; 3 Global Blood Therapeutics, Inc., South San Francisco, California, United States of America; 4 Division of Hematology, Children’s Hospital of Philadelphia, Philadelphia, Pennsylvania, United States of America; University of Illinois at Chicago, UNITED STATES

## Abstract

**Background:**

Among individuals with sickle cell disease (SCD), decreased hemoglobin is associated with lower oxygen saturation (SpO_2_) and increased risk of stroke, both of which are associated with lower intelligence quotient (IQ) scores. Thus, increasing hemoglobin and SpO_2_ in individuals with SCD may increase IQ and educational attainment.

**Methods:**

A cohort simulation model was built to determine academic performance and educational attainment based on cognitive function (measured by IQ) of a pediatric SCD cohort randomly assigned to treatment and control groups. The model contained two key stages: childhood (<10 years) and adolescence (≥10 years). In stage 1, increased hemoglobin and increased SpO_2_ (assigned to the treatment group) were determinants of higher IQ, prevention of IQ deterioration over time. Increased hemoglobin was also a determinant of decreased stroke risk. In stage 2, improvement in adolescent IQ as a result of treatment was a determinant of academic performance.

**Results:**

In a simulated cohort of 2000 children and adolescents with SCD (52.5% female, 50% treated), stroke incidence was predicted to be 44.4% lower among the treated group than the untreated group (4.5% versus 8.1%, respectively). The average IQ among the treated group was estimated to be 91.1 compared with 82.9 in the untreated group (a 9.9% difference; *P*<0.001). Finally, high school (≥12 years of education) completion rates were estimated to be 64.7% higher among the treated group: 76.1% of the treated group was projected to complete high school compared with 46.2% of the untreated group.

**Conclusions:**

Our model predicts that an average improvement in hemoglobin of 1.1 g/dL (11 g/L) among individuals with SCD may be associated with improved neurocognition and educational outcomes. These improvements may also generate benefits not captured by our model, including improved quality of life, employment, and income.

## Introduction

Patients with sickle cell disease (SCD) experience lifelong symptoms and complications, such as hemolytic anemia, vaso-occlusive crises, organ damage, and leg ulcers, and they are at an increased risk for life-threatening conditions, such as stroke and silent cerebral infarct. Although the prevalence of SCD in the United States is not precisely known, it is estimated to affect approximately 100,000 individuals, most of whom are African American or Hispanic; SCD is associated with higher rates of unemployment and lower family incomes [1–3]. A recent cohort simulation model comparing patients with SCD to the unaffected population reported a 22-year lower life expectancy and a $695,000 difference in lifetime income [3]. An estimated 50% of patients with SCD do not survive beyond 50 years of age, with common causes of death including infection, chest syndrome, and stroke [4, 5]. Approximately 1 in 4 patients with the most severe form of SCD, sickle cell anemia (homozygous inheritance of hemoglobin S [HbS] or the HbSS genotype), experience a stroke by age 45 [6, 7]. Aside from overt stroke, the most common cerebrovascular accident (CVA) among patients with SCD is silent cerebral infarct, which is characterized by abnormal magnetic resonance imaging results despite no prior history of CVA and are associated with decreased cognitive functioning [8, 9].

The reduced oxygen affinity of HbS and altered rheology of blood in patients with SCD can cause pulmonary hypertension, reduced tissue perfusion, and decreased intellectual functioning, which is associated with academic achievement. The effects of chronically reduced oxygen delivery suggest that increasing hemoglobin (Hb) levels and/or oxygen saturation (SpO_2_) in patients with SCD could improve intellectual function and academic achievement [10].

While stem cell transplant remains the only curative option for patients with SCD [11], presently available therapies, including blood transfusion, hydroxyurea, and voxelotor have been shown to increase Hb in both children and adults [12, 13]. Voxelotor is a first-in-class HbS polymerization inhibitor that directly increases Hb by inhibiting the root cause of SCD—polymerization of deoxygenated HbS—thereby reducing red blood cell (RBC) destruction. In a clinical study by Hood et al, patients with SCD achieved better cognitive functioning test results ≤3 days after a blood transfusion, at which point Hb levels are higher, compared with those 3 to 7 weeks after a blood transfusion [14]. To date, preliminary data are available for the effects of hydroxyurea on neurocognition in children with SCD. A prospective clinical pilot study of 29 children (58% female) with sickle cell anemia who received hydroxyurea treatment for 1 year demonstrated improvement in reading passage comprehension [15]. Significant positive associations were also observed between Hb and working memory; however, the primary hypothesis of lowered cerebral blood flow was not reached in this study. In another study, 15 children with SCD on hydroxyurea for at least 1 year achieved significantly higher test results for verbal comprehension, fluid reasoning, and general cognitive ability than 50 children with SCD not using hydroxyurea after controlling for demographics and hematocrit [16]. Overall, there are a lack of studies demonstrating the robust potential for SCD treatment to improve cognitive function in childhood and affect educational attainment among patients with SCD.

Despite evidence that increasing Hb and SpO_2_ in individuals with SCD may increase their intelligence quotient (IQ) [9], data that link IQ, academic performance, and educational attainment to Hb-increasing SCD treatment are limited. In this study, we built a cohort simulation model to estimate how potential improvements in cognitive function (measured by IQ) through Hb-improving SCD treatment beginning in childhood can affect academic performance and achievement and educational attainment among patients with SCD in the United States.

## Methods

A cohort simulation model was built to reflect the pediatric SCD population and used to estimate how improvements in pediatric cognitive function, as measured by IQ, affect academic performance and educational attainment. Cognitive function was measured in randomized cohorts receiving treatment for SCD. The pediatric SCD cohort was designed to have a comparable sex and race/ethnicity (African American race and Hispanic ethnicity) composition to real-world individuals with SCD. All results were calculated separately for each group (treated vs untreated) and by sex. This study did not require institutional review board approval or informed consent because it relied solely on deidentified publicly available data and published literature.

### Model development

**Fig 1:**
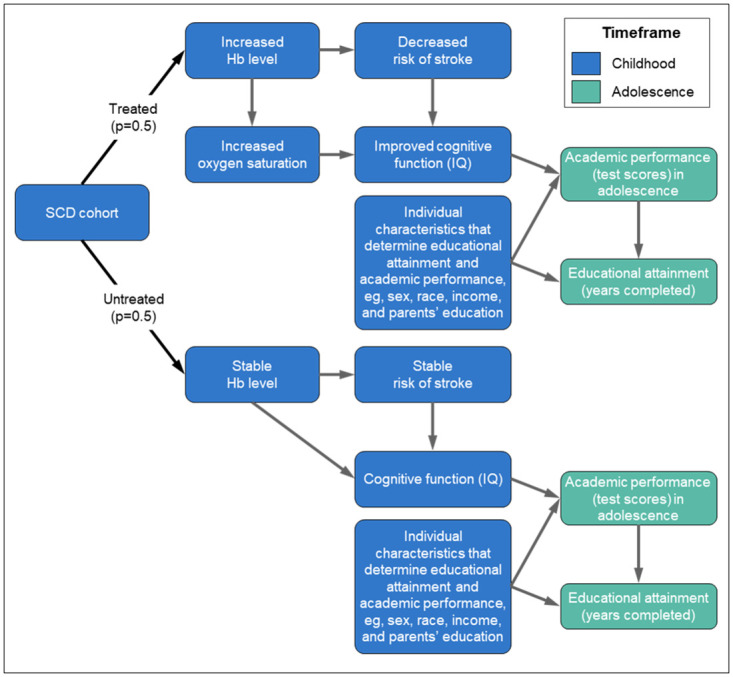
Model diagram. Hb, hemoglobin; IQ, intelligence quotient; SCD, sickle cell disease.

Model parameter sources were identified in the clinical and economic literature and selected based on their representativeness of the SCD patient population, recentness, and relevance to the model framework (S1 Table). The HOPE-KIDS 1 trial (NCT02850406) was not finalized at the time of model development and was therefore not included as a source. Three main topic areas were explored: (i) SCD patient cohort characteristics, including IQ, risk of stroke/silent infarct, academic performance, and noncognitive skills (i.e., social skills, self-esteem, self-control, motivation); (ii) the links between anemia, Hb, SpO_2_, and cognitive function/IQ and risk of stroke; and (iii) the links between cognitive function/IQ, academic performance/achievement, and educational attainment. Key model parameters are summarized in Table 1. The model calculated the educational attainment of members of a pediatric SCD patient cohort who were randomly assigned to treatment and control groups. The model has two key stages: (1) childhood (<10 years) and (2) adolescence (≥10 years). The model framework is shown in Fig 1.

**Table 1:** Key model parameter values.

Parameter	Symbols	Value	Sources
**Treatment effect on hemoglobin** ^a^	Δ*Hb*_*t*_	1.1 g/dL increase (95% CI: 0.9–1.4)	Vichinsky et al. (2019) [13]
**Treatment effect on SpO_2_**	Δ*O2*_*t*_	3.10 percentage point increase per 1 g/dL increase in Hb	Blyden et al. (2018) [17]
**Relative stroke risk (infarctive)**	*r*	1.85 (95% CI: 1.32–2.59) per 1 g/dL decrease in Hb	Ohene-Frempong et al. (1998) [7]
**Reduction in IQ score in stage 1 resulting from stroke**	*k*_s_	–15.86	Kawadler et al. (2016) [18]
**Reduction in IQ score in untreated state between stages 1 and 2 independent of stroke/infarct status**	*k*_*c*_	–5.00	Wang et al. (2001) [19]
			King et al. (2014) [9]
**Increase in IQ score per 1 percentage point increase in SpO2**	*K*_*t*_	0.75	King et al. (2014) [9]
**Baseline initial IQ**	IQ_0_	Mean 89.18 (95% CI: 86.36–92.00)	Kawadler et al. (2016) [18]
**Baseline initial stroke risk**	*S*_0_	Mean 7.20% (SD 0.7)	DeBaun et al. (2012) [8]
**Probability of being female**	*p*_*f*_	52.00%	Farber (1985) [2]
**Probability of attending preschool**	*Preschool*	32.00%	Farber (1985) [2]
**AFQT score distribution**	*σ*_*AFQT*_, *μ*_*AFQT*_	Mean 25.15 (SD 21.97)	NLSY79 [20]
**Mother’s years education completed**	*Mother*	Mean (SD)	NLSY79 [20]
**Father’s years education completed**	*Father*	Mean (SD)	NLSY79 [20]
**Social skill**	*Social*	Socialization variable takes the value 1 if score ≥3, and 0 otherwise. Mean 0.37 (SD 0.48)	NLSY79 [20], Heckman and Raut (2016) [21]
**Self-esteem score (Rosenberg scale)**	*Self-esteem*	Mean 31.21 (SD 5.45). Self-esteem variable takes the value 1 if score ≥20, and 0 otherwise	Burlew (2000) [22], Heckman and Raut (2016) [21]
**Internal self-concept (Pearlin scale)**	*Self-concept*	Self-concept variable takes the value 1 if Pearlin score ≥23, and 0 otherwise. Mean 0.40 (SD 0.49)	NLSY79 [20], Heckman and Raut (2016) [21]
**Job aspiration/motivation score**	*Motivation*	Motivation variable takes the value 1 if average motivational score ≥3.75, and 0 otherwise. Mean 0.56 (SD 0.50)	NLSY79 [20], Heckman and Raut (2016) [21]

^a^For hemoglobin: the conversion factor from conventional units (g/dL) to SI units (g/L) is 10.

AFQT, Armed Forces Qualification Test; CI, confidence interval; IQ, intelligence quotient; NLSY79, National Longitudinal Survey of Youth 1979; SD, standard deviation; SpO_2_, oxygen saturation.

### Treatment effect pathways

In the first stage, the treatment group was assigned a rise in Hb and SpO_2_ levels, which affected IQ independently and directly—increasing it and preventing the deterioration of IQ over time—and indirectly by decreasing the risk of stroke. This treatment effect pathway is based on studies that showed a significant reduction in the relative risks of infarctive and hemorrhagic stroke per 1 g/dL (10 g/L) increase in Hb (Ohene-Frempong et al) and increase in full-scale intelligence quotient (FSIQ) per 1% absolute increase in SpO_2_ (King et al) [7, 9]. Although it is possible that stroke directly affects educational attainment and academic achievement, there was no evidence in the literature to inform and model this linkage in our model. The only study identified that examined differences in educational attainment for SCD patients with history of stroke versus those without did not find a significant difference in rates of high school graduation or being on track to graduate [23].

Among untreated patients in the model, IQ was assumed to deteriorate between childhood and adolescence, as found in several studies showing IQ deterioration between childhood and adolescence among patients with SCD that was independent of stroke/silent infarct/CVA status [19, 24]. The modeled treatment effects on Hb and SpO_2_, Δ*Hb*_*t*_ and Δ*O2*_*t*_, were based on results from the phase 3, randomized, placebo-controlled Hemoglobin Oxygen Affinity Modulation to Inhibit HbS Polymerization (HOPE) trial of voxelotor in patients 12 to 65 years of age with SCD and from the literature [13]. Voxelotor was granted accelerated approval by the US Food and Drug Administration in November 2019 for the treatment of SCD in patients aged 12 years and older following the HOPE trial and was approved in December 2021 for patients aged 4 years and older [25, 26]. Voxelotor has also been approved for the treatment of patients with SCD aged 4 years and older in the United Arab Emirates, and aged 12 years and older in Oman, Kuwait, and Saudi Arabia [27]. In the European Union and Great Britain voxelotor is approved as monotherapy or in combination with hydroxyurea for the treatment of hemolytic anemia in patients with SCD aged 12 years and older [28, 29].

### Academic performance and educational attainment

In the second stage, IQ was assigned as a determinant of academic performance and achievement, as measured by Armed Forces Qualification Test (AFQT) scores, and educational attainment, as measured by years of education completed, which are determined by individual characteristics (including noncognitive skills) and AFQT scores. Specifically, the modeled relationship between IQ and AFQT scores was based on the study by Borghans et al that estimated the relationship between IQ z scores and AFQT z scores using the National Longitudinal Survey of Youth 1979 (NLSY79) data [30].

The determinants of years of education completed, including both cognitive and noncognitive skills among other factors, were based on a wide range of economic studies that have identified such factors [21, 31–35]. The specific parameter values for the contribution of each of the individual characteristics and AFQT scores came from a study by Heckman and Raut [21] that estimated the effect of participating in preschool programs on cognitive skills (measured by AFQT scores) in children with low socioeconomic status; noncognitive skills (i.e., self-esteem, socialization, motivation, and self-control); years of education completed; and average annual earnings. Specifically, one component of their analysis involved examining the contribution of cognitive skill and AFQT score to years of education completed, accounting for the mother’s and father’s education, noncognitive skills, and whether the individual attended preschool (Table 2) [21].

**Table 2:** Cohort simulation model results.

Outcome	Group		Difference		
	Untreated	Treated	Absolute	Percentage	*P*-value
	**Panel A: Baseline**				
**Stroke incidence**	8.1%	4.5%	–3.6%	–44.4%	0.001
**IQ score**	82.85	91.08	8.23	9.9%	<0.001
**Years of education**	11.87	12.53	0.65	5.5%	<0.001
**% completing ≥12 years of education**	46.2%	76.1%	29.9%	64.7%	<0.001
	**Panel B: Scenario 1**				
**Stroke incidence**	8.3%	4.3%	–4.0%	–48.2%	<0.001
**IQ score**	82.72	91.03	8.30	10.0%	<0.001
**Years of education**	11.50	12.17	0.66	5.8%	<0.001
**% completing ≥12 years of education**	18.7%	65.0%	46.3%	247.6%	<0.001
	**Panel C: Scenario 2**				
**Stroke incidence**	6.9%	4.9%	–2.0%	–29.0%	0.058
**IQ score**	82.96	90.94	7.99	9.6%	<0.001
**Years of education**	11.86	12.55	0.69	5.8%	<0.001
**% completing ≥12 years of education**	44.9%	75.2%	30.3%	67.5%	<0.001

IQ, intelligence quotient.

### Statistical analysis

In stage 1 of the model, SCD treatment augmented IQ directly through SpO_2_ and indirectly through Hb and risk of stroke. Specifically, every 1 g/dL (10 g/L) increase in Hb generated from treatment decreased the relative risk of CVA (Equation e1 in S1 File) [7].

Stage 2 of the model took IQ at the end of stage 1 (Equations e2 and e3 in S2 File) as an input to determine academic performance based on educational attainment, as measured by years of education completed (Equation e4 in S3 File) and AFQT score (Equations e5-e8 in S4 File) [21, 30].

The National Longitudinal Survey of Youth 1979 (NLSY79) is a publicly available, nationally representative sample of 12,686 young men and women born between 1957 and 1964 living in the United States in 1979. Respondents were between the ages of 14 and 22 when first surveyed in 1979 and are re-surveyed annually. Information on participants’ family background, income, education, health, children, crime history, and employment is collected. Information on SCD patients’ fathers’ years of education completed, socialization skills, self-concept skill, and motivation skills could not be identified in the literature; therefore, values for these variables were based on averages for low-income African Americans in the NLSY79 [20]. The data set from the NLSY79 was chosen because they were the data used by Heckman and Raut to estimate determinants of educational attainment and earnings and because the patient population has similar sociodemographic background characteristics to those of the SCD patient population. These values were estimated among respondents reporting Black or Hispanic race and income below the poverty line at the initial interview (1979). The present analysis was completed in Stata-MP version 16.0 (StataCorp LLC).

### Scenario analysis

Two scenario analyses were conducted to (i) isolate the impact of SCD treatment on educational outcomes and (ii) explore the potential impact of the effect of treatment on noncognitive skills, which in turn impact educational outcomes. First, we considered a scenario in which all patients in the cohort had the same baseline levels of socioeconomic variables, including noncognitive skills, mother’s education, father’s education, and preschool attendance. This scenario isolated the impact of cognitive skills on educational attainment. Second, we considered the scenario in which treatment impacts development of noncognitive skills as well as cognitive skills. Specifically, we modeled the scenario in which treatment increased the probability that the socialization, self-concept, and motivation noncognitive skills take a value of 1 by 10%. This translates to an increase in the probability of having high socialization, self-concept, and motivation noncognitive skills of 0.0368, 0.0397, and 0.0557, respectively. For self-esteem, we modeled an increase in self-esteem of 10%, that is, 3.12 points on the Rosenberg scale, among treated individuals.

## Results

**Fig 2:**
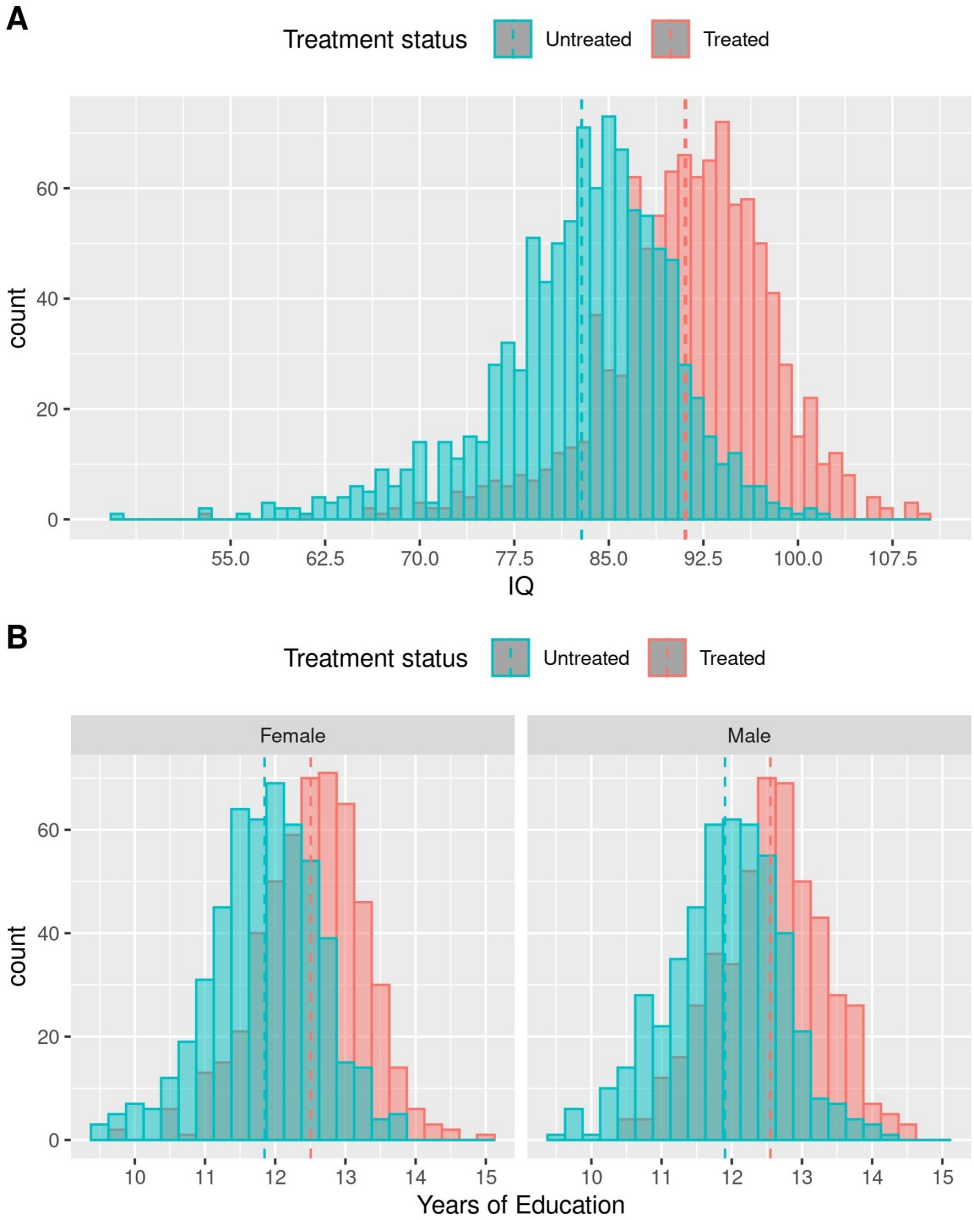
Distribution of IQ score (A) and years of education completed (B) by treatment status (A, B) and sex (B). IQ, intelligence quotient.

In the baseline analysis, among a simulated cohort of 2000 pediatric patients with SCD (51.5% female, 50% treated, 50% untreated), the incidence of stroke was predicted to be 44.4% lower among the treated group in the model compared with the untreated group (*p* = 0.001; Table 2). In the baseline analysis, 4.5% of those in the treated group would have a stroke versus 8.1% of those in the untreated group. The average IQ score among those treated was estimated to be 91.1, compared with 82.9 among the untreated group, a difference of 9.9% (*p*<0.001; Fig 2A).

Years of education completed was estimated to be 5.5% higher among the treated group than the untreated group, with those in the treated group projected to finish 12.5 years of education on average compared with 11.9 years of school completed on average for the untreated group (*p*<0.001). This would translate to high school completion rates (≥12 years of education completed) that are 64.7% higher among the treated group than the untreated group (*p*<0.001); 76.1% of the treated group compared with 46.2% of the untreated group were projected to complete ≥12 years of education (Table 2 and Fig 2B). On average, the number of years of education completed among men would be similar to that among women in both the treated (12.6 vs 12.5 years, respectively) and untreated groups (11.8 vs 11.9 years, respectively, *p*<0.001).

In the first scenario, where all patients in the cohort were adjusted to the same baseline levels by socioeconomic variables—including noncognitive skills, mother’s education, father’s education, and preschool attendance—the incidence of stroke, IQ, years of education completed, and high school completion rates were all estimated to be significantly improved in the treatment group (all *p*<0.001). In particular, the high school completion rate among the treated group (65.0%) was more than double that of the untreated group (18.7%). In the second scenario analysis, in which treatment also improved noncognitive skills, IQ, years of education completed, and high school completion rates were all predicted to be significantly improved in the treatment group (all *P*<0.001). The modeled impact of treatment on educational attainment through noncognitive skills was relatively modest, increasing the difference in years of education completed between the treated and untreated groups to 5.8% versus 5.5% in the baseline analysis.

## Discussion

Overall, complications of SCD present a multitude of challenges in terms of disease symptoms and cognitive and educational disadvantages throughout the lifespan. Studies have documented absenteeism ranging from 1 to 30 missed school days in a single year for children with SCD, which likely takes a toll on academic achievement and attainment [36–39]. Frequent vaso-occlusive episodes (VOEs) can be a prime reason for school absenteeism owing to hospitalizations or management at home if barriers to care exist [40]. Multimodal therapy to improve anemia, VOEs, and their sequelae remains an unmet need, which if addressed has the potential to mitigate the burden of cognitive challenges related to organ dysfunction and increased morbidities. As one of the major complications in children with SCD, cerebral injuries such as overt strokes and silent cerebral infarcts have been shown to impose severe cognitive deficits that significantly affect academic performance (reading and math scores) and age- and gender-matched IQs compared with children who have SCD but no history of a CVA [41–43]. Deficits in academic attainment are evident from an early age, with low preschool enrollment, and extend to a higher likelihood of grade retention, lower rate of high school completion, and requirement of special education services [2, 44, 45].

A large body of economic research has demonstrated that educational attainment in children has a consequential role in income achievement during adult life [21, 46]. A recent cohort simulation model estimated sizeable reductions in longevity and lifetime income attainment specifically among those with SCD [3]. Although our model does not capture benefits of treatment such as improved quality of life, employment, and income among individuals with SCD, it is reasonable to assume that the modeled improvements in cognitive function and increased educational attainment would also generate improvements in employment and income.

This analysis demonstrates that children with SCD could have better cognitive function (IQ) and lower risk of stroke by increasing Hb and SpO_2_. Our model predicts that a treatment that results in an average improvement in Hb of 1.1 g/dL (11 g/L) may be associated with a significantly lower rate of stroke, significantly increased IQ, and significantly more years of education completed [13]. Specifically, we estimate that an average 1.1 g/dL (11 g/L) improvement in Hb could reduce the incidence of stroke by 44.4%, increase IQ by nearly 9.9%, and increase years of education completed by 5.5%. To our knowledge, this study is the first to model the potential improvements in cognitive function and educational attainment attributable to improvements in Hb and SpO_2_ among pediatric patients with SCD in the United States.

In our baseline analysis, 4.5% of those in the treated group experienced a stroke versus 8.1% of those in the untreated group, representing a greater than 40% reduction in the incidence of stroke with treatment that increases Hb and improves SpO_2_. This implies that improvements in Hb levels from innovative treatments, in combination with transcranial Doppler ultrasonography screening and RBC transfusion therapy [47, 48], could reduce the incidence of stroke in pediatric patients with SCD.

As more data on this topic emerges, future analyses could include an adaptation of our model to examine the cognition and education effects on patients by Hb genotype (e.g., HbSS, Hb heterozygosity [HbSC], sickle-β0-thalassemia [HbSβ0], sickle-β+-thalassemia [HbSβ+], and hereditary persistence of fetal hemoglobin [HPHF]).

### Limitations

This study has some limitations. The model assumes that treatment prevents deterioration of IQ and will increase educational attainment and academic achievement in the treated group relative to the untreated group, where it is assumed that IQ deteriorates over time. If IQ does not deteriorate in untreated individuals or deteriorates at a slower rate than that observed in previous studies [9, 19, 24], then our model understates educational attainment in the untreated population. The analysis in the model assumes that an improvement in IQ is irrespective of baseline Hb levels, or that all children have the same mean baseline Hb level at cohort entry. This overlooks the variability in Hb levels among patients with SCD and the different clinical outcomes associated with changes in Hb levels. The baseline analysis also assumes that treatment does not affect noncognitive skills. Thus, to the extent that treatment would actually increase noncognitive skills, our baseline model will understate the impact of treatment on educational attainment. Additionally, our model only captures the impact of stroke on academic achievement and educational attainment through IQ and did not model grade retention or utilization of special education services. To the extent that stroke directly impacts educational attainment and academic achievement, for example, by increasing the likelihood that a student drops out of school, our model understates the impact of treatment. Our model also does not capture other cerebrovascular events such as silent infarct and transient ischemic attack; it only captures overt stroke. Absenteeism from school could also be explored more thoroughly because failure to attain educational goals may not be directly related to a lower IQ in all situations.

In addition, several model parameters, including fathers’ years of education completed, socialization skills, self-concept skills, and motivation skills of patients with SCD, could not be identified in the literature or for the SCD patient population specifically; therefore, values for these parameters were estimated using NLSY79 data for non-White individuals. These data are relatively old and may not be representative of the current pediatric SCD patient population’s noncognitive skills or the educational attainment of SCD patients’ parents. While it may be plausible that treatment may also improve noncognitive skills directly and/or indirectly though greater educational attainment, we did not include this mechanism of action in our model. To that extent, our estimates understate the benefits of treatment.

## Conclusion

As demonstrated in this analysis, children with SCD with increased Hb and SpO_2_ have better cognitive function (measured by IQ) and lower risk of stroke. Our model predicts that treatment resulting in an average improvement in Hb of 1.1 g/dL (11 g/L) may be associated with improved neurocognition and educational outcomes. These improvements may also generate benefits not captured by our model, including improved quality of life, employment, and income among individuals with SCD.

## Supporting information

S1 Table Targeted literature search strategy. (PDF)

S1 File Equation e1. Probability of stroke as a function of receipt of treatment.
(PDF)

S2 File Equations e2 and e3. IQ at the end of stage 1 in treated and untreated/control individuals.
(PDF)

S3 File Equation e4. Years of education completed.
(PDF)

S4 File Equation e5-8. AFQT score as a function of standardized IQ score.
(PDF)
