# Supplementary material for: Cognition and education benefits of increased hemoglobin and blood oxygenation in children with sickle cell disease
Source: PLoS One. 2023 Aug 8;18(8):e0289642. doi: 10.1371/journal.pone.0289642 (PMC10409269; doi:10.1371/journal.pone.0289642)
Supplement: S1 Table — (PDF) [file pone.0289642.s001.pdf]

Supporting information

Cognition and education benefits of increased hemoglobin and blood oxygenation in children with sickle cell disease

Joanna P. MacEwan*, Allison A. King, Andy Nguyen,

Anuj Mubayi, Irene Agodoa, Kim Smith-Whitley

***Corresponding author:** jmacewan@genesisrg.com (JPM)

Table of Contents

Targeted literature search strategy………………………………………………………………………............ 2

Table s1. Targeted literature search strategy.

Order	Database	Description	Search terms
1	PubMed	All terms (population, hemoglobin, cognition, education)	(“Anemia, Sickle Cell”[MeSH]) AND (“Hemoglobins”[MeSH] or "anemia"[MeSH]) AND (“Cognition”[MeSH] or “Cognitive dysfunction”[MeSH] or “Schools”[MeSH] or “educational status”[MeSH] or “academic performance"[MeSH] or “academic success”[MeSH])
2	Google Scholar	Google Scholar Part 1 (cognitive function)*	(“sickle cell disease” OR “sickle cell anemia”) AND ("deoxygenated" OR “hypoxia” OR “hypoxemia”) AND (“cognitive function” OR “cognitive ability” OR “cognitive impairment”) -mice
3	Google Scholar	Google Scholar Part 2 (education)*	(“sickle cell disease” OR “sickle cell anemia”) AND ("deoxygenated" OR “hypoxia” OR “hypoxemia”) AND (“educational attainment” OR “academic performance” OR “academic achievement”)

*Two separate Google Scholar searches were conducted to accommodate search field character limits.
