# Supplementary material for: Cognition and education benefits of increased hemoglobin and blood oxygenation in children with sickle cell disease
Source: PLoS One. 2023 Aug 8;18(8):e0289642. doi: 10.1371/journal.pone.0289642 (PMC10409269; doi:10.1371/journal.pone.0289642)
Supplement: S1 File — Probability of stroke as a function of receipt of treatment. (PDF) [file pone.0289642.s002.pdf]

Supporting information

Cognition and education benefits of increased hemoglobin and blood oxygenation in children with sickle cell disease

Joanna P. MacEwan*, Allison A. King, Andy Nguyen,
Anuj Mubayi, Irene Agodoa, Kim Smith-Whitley

***Corresponding author:** jmacewan@genesisrg.com (JPM)

Table of Contents

Equation e1. Probability of stroke as a function of receipt of treatment........ 2

References........ 2

Equation e1. Probability of stroke as a function of receipt of treatment

$$S_t = \Pr(stroke|\text{treated}) = r^{-1}\Delta Hb_t S_0, \quad \text{(e1)}$$

where ΔHb_t denotes every 1 g/dL (10 g/L) increase in hemoglobin (Hb) generated from treatment in stage 1, S_0 denotes the relative risk of cerebrovascular accident (CVA), and every 1 g/dL (10 g/L) increase in ΔHb_t decreased the relative risk of CVA from S_0 to $r^{-1}S_0$ percent [1]. t denotes receipt of treatment, and every 1 percentage point increase in Hb-oxygen saturation generated from treatment $\Delta 02_t$ in stage 1 increased intelligence quotient (IQ) directly by k_t points and also prevented deterioration of IQ between stage 1 and 2. In untreated individuals, IQ deteriorated by k_c (ie, $k_c < 0$) points by the end of stage 1. The rate of deterioration was not related to or independent of infarct status [2-4].

References

1. Ohene-Frempong K, Weiner SJ, Sleeper LA, Miller ST, Embury S, Moohr JW, et al. Cerebrovascular accidents in sickle cell disease: rates and risk factors. Blood.1998;91:288–294.
2. Steen R, Fineberg-Buchner C, Hankins G, Weiss L, Prifitera A, Mulhern RK. Cognitive deficits in children with sickle cell disease. J Child Neurol. 2005;20:102–107.
3. Wang W, Enos L, Gallagher D, Thompson R, Guarini L, Vichinsky E, et al. Neuropsychologic performance in school-aged children with sickle cell disease: a report from the Cooperative Study of Sickle Cell Disease. J Pediatr. 2001;139:391–397.
4. King AA, Strouse J, Rodeghier M, Compas BE, Casella JF, McKinstry RC, et al. Parent education and biologic factors influence on cognition in sickle cell anemia. Am J Hematol. 2014;89:162–167.
