# Supplementary material for: Cognition and education benefits of increased hemoglobin and blood oxygenation in children with sickle cell disease
Source: PLoS One. 2023 Aug 8;18(8):e0289642. doi: 10.1371/journal.pone.0289642 (PMC10409269; doi:10.1371/journal.pone.0289642)
Supplement: S2 File — IQ at the end of stage 1 in treated and untreated/control individuals. (PDF) [file pone.0289642.s003.pdf]

Supporting information

Cognition and education benefits of increased hemoglobin and blood oxygenation in children with sickle cell disease

Joanna P. MacEwan*, Allison A. King, Andy Nguyen,
Anuj Mubayi, Irene Agodoa, Kim Smith-Whitley

***Corresponding author:** jmacewan@genesisrg.com (JPM)

Table of Contents

Equations e2 and e3. IQ at the end of stage 1 in treated (e2) and untreated/control (e3) individuals.......... 2

References.. 2

Equations e2 and e3. IQ at the end of stage 1 in treated (e2) and untreated/control (e3) individuals.

Stroke reduced IQ in stage 1 by k_s (ie, $k_s < 0$) points in both the untreated and treated groups [1]. Thus, in treated patients, IQ at the end of stage 1, $IQ_{t,1,i}$, is given by:

$$IQ_{t,1,i} = IQ_{t,0,i} + k_t \Delta 02_t + 1(\text{stroke}_t) \times k_s, \qquad \text{(e2)}$$

and untreated/control individuals (denoted with subscript c), IQ at the end of stage 1, $IQ_{c,1,i}$, is given by:

$$IQ_{c,1,i} = IQ_{c,0,i} + k_c + 1(\text{stroke}_c) \times k_s. \qquad \text{(e3)}$$

References

1. Wang W, Enos L, Gallagher D, Thompson R, Guarini L, Vichinsky E, et al. Neuropsychologic performance in school-aged children with sickle cell disease: a report from the Cooperative Study of Sickle Cell Disease. J Pediatr. 2001;139:391–397.
