# Supplementary material for: Cognition and education benefits of increased hemoglobin and blood oxygenation in children with sickle cell disease
Source: PLoS One. 2023 Aug 8;18(8):e0289642. doi: 10.1371/journal.pone.0289642 (PMC10409269; doi:10.1371/journal.pone.0289642)
Supplement: S3 File — Years of education completed. (PDF) [file pone.0289642.s004.pdf]

Supporting information

Cognition and education benefits of increased hemoglobin and blood oxygenation in children with sickle cell disease

Joanna P. MacEwan*, Allison A. King, Andy Nguyen,
Anuj Mubayi, Irene Agodoa, Kim Smith-Whitley

***Corresponding author:** jmacewan@genesisrg.com (JPM)

Table of Contents

Equation e4. Years of education completed 2

References 2

Equation e4. Years of education completed.

$$e_i = \beta_0 + \beta_1 mom_i + \beta_2 dad_i + \beta_3 preschool_i + \beta_4 AFQT_i + \beta_5 soc_i + \beta_6 motive_i + \beta_7 esteem_i + \beta_8 control_i, \quad \text{(e4)}$$

where mom_i is mother's years of education completed, dad_i is father's years of education completed, $preschool_i$ is a binary indicator for having attended any preschool, soc_i is a binary indicator for higher social skills (corresponding to a score of 3-4 on self-assessed sociability), $motive_i$ is a binary indicator for above-average motivational skill (corresponding to an average motivational skill score >3.75), $esteem_i$ is a binary indicator for high self-esteem (corresponding to a score of >20 on the Rosenberg scale), and $control_i$ is a binary indicator for high self-control (corresponding to a score of 23-28 on the Pearlin Mastery scale of internal concept) [1].The model was built in Microsoft Excel.

References

1. Heckman JJ, Raut LK. Intergenerational long-term effects of preschool - structural estimates from a discrete dynamic programming model. J Econom. 2016;191:164–175.
