# Supplementary material for: Cognition and education benefits of increased hemoglobin and blood oxygenation in children with sickle cell disease
Source: PLoS One. 2023 Aug 8;18(8):e0289642. doi: 10.1371/journal.pone.0289642 (PMC10409269; doi:10.1371/journal.pone.0289642)
Supplement: S4 File — AFQT score as a function of standardized IQ score. (PDF) [file pone.0289642.s005.pdf]

Supporting information

Cognition and education benefits of increased hemoglobin and blood oxygenation in children with sickle cell disease

Joanna P. MacEwan*, Allison A. King, Andy Nguyen,
Anuj Mubayi, Irene Agodoa, Kim Smith-Whitley

***Corresponding author:** jmacewan@genesisrg.com (JPM)

Table of Contents

Equations e5-8. AFQT score as a function of standardized IQ score. 2

References........ 2

Equations e5-8. AFQT score as a function of standardized IQ score.
Stage 2 of the model takes IQ at the end of stage 1, $IQ_{1,i}$, as an input to determine academic performance measured by Armed Forces Qualification Test (AFQT) score, $AFQT_i$, and education attainment, as measured by years of education completed, e_i. [1,2] Specifically, standardized IQ score (IQ z score) was used to predict standardized AFQT score (AFQT z score):

$$AFQTz_i = \alpha_0 + \alpha_1 IQz_i, \tag{e5}$$

where

$$IQz_i = \frac{IQ_i - \mu_{IQ}}{\sigma_{IQ}}, \tag{e6}$$

and

$$AFQTz_i = \frac{AFQT_i - \mu_{AFQT}}{\sigma_{AFQT}}. \tag{e7}$$

AFQT scores were calculated as:

$$AFQT_i = \sigma_{AFQT} AFQTz_i + \mu_{AFQT}. \tag{e8}$$

References

1. Heckman JJ, Raut LK. Intergenerational long-term effects of preschool - structural estimates from a discrete dynamic programming model. J Econom. 2016;191:164–175.
2. Borghans L, Golsteyn BH, Heckman JJ, Humphries JE. What grades and achievement tests measure. Proc Natl Acad Sci U S A. 2016;113:13354–13359.
